# Transgenic Cabbage Expressing Cry1Ac1 Does Not Affect the Survival and Growth of the Wolf Spider, *Pardosa astrigera* L. Koch (Araneae: Lycosidae)

**DOI:** 10.1371/journal.pone.0153395

**Published:** 2016-04-07

**Authors:** Young-Joong Kim, Joon-Ho Lee, Chee Hark Harn, Chang-Gi Kim

**Affiliations:** 1 Bio-Evaluation Center, Korea Research Institute of Bioscience and Biotechnology, Cheongju 28116, Republic of Korea; 2 Entomology program, Department of Agricultural Biotechnology, Seoul National University, Seoul 08826, Republic of Korea; 3 Research Institute of Agriculture and Life Sciences, Seoul National University, Seoul 08826, Republic of Korea; 4 R&D Headquarters, Nongwoo Bio Co., Yeoju 12648, Republic of Korea; French National Institute for Agricultural Research (INRA), FRANCE

## Abstract

Both herbivores that consume transgenic crops and their predators can be exposed to insecticidal proteins expressed in those crops. We conducted a tritrophic bioassay to evaluate the ecotoxicological impacts that *Bt* cabbage (*Brassica oleracea* var. *capitata*) expressing Cry1Ac1 protein might have on the wolf spider (*Pardosa astrigera*), a non-target generalist predator. Enzyme-Linked Immunosorbent Assays indicated that protein levels were 4.61 ng g^-1^ dry weight in fruit flies (*Drosophila melanogaster*) fed with the transgenic cabbage and 1.86 ng g^-1^ dry weight in the wolf spiders that preyed upon them. We also compared the life history traits of spiders collected from *Bt* versus non-*Bt* cabbage and found no significant differences in their growth, survival, and developmental rates. Because *Bt* cabbage did not affect the growth of fruit flies, we conclude that any indirect effects that this crop had on the wolf spider were probably not mediated by prey quality. Therefore, exposure to Cry1Ac1 protein when feeding upon prey containing that substance from transgenic cabbage has only a negligible influence on those non-target predatory spiders.

## Introduction

The conventional use of transgenic *Bacillus thuringiensis* (*Bt*) crops that express insecticidal δ-endotoxins (Cry proteins) as an anti-pest agent has greatly increased since 1996 [1]. Adoption of these *Bt* crops has helped reduce the amount of damage from targeted pests and the cost of insecticide use [2–4]. However, the potential influence of insect-resistant transgenic crops on non-target organisms, including primary consumers and predators, has been an important issue when assessing possible environmental risks from those plants. For example, *Bt* toxins can be transferred not only directly to crop-fed herbivores but also indirectly to their predators via trophic pathways [5]. Therefore, risk assessments have been conducted to address concerns about environmental safety and any negative effects of *Bt* crops on the non-target food chain [6,7].

Among the many important predator groups found in agricultural ecosystems, spiders are the most ubiquitous generalist predators, playing an important role in regulating insect pest populations [8]. Spiders can be exposed to *Bt* toxin through various ecological routes. For example, they ingest pollen trapped in web silk [9] or else feed on pollen-dusted prey [10] or herbivores that have consumed *Bt* crops [11–14]. Several studies have assessed the effects of those crops on the abundance of spiders in fields [15]. However, well-controlled tritrophic bioassays under laboratory conditions are also necessary [16,17]. Reports have been mixed about the possible interactions between engineered crops and spider health. For example, transgenic rice that expresses Cry1Ab protein has been examined with such bioassays to determine whether those plants influence species within Lycosidae (*Pardosa pseudoannulata*) and Linyphiidae (*Ummeliata insecticeps*) that prey upon planthoppers (*Nilaparvata lugens*; Homoptera: Delphacidae) and rice leafrollers (*Cnaphalocrocis medinalis*; Lepidoptera: Pyralidae) [12–14]. Those examinations have revealed that the Cry1Ab and Cry3Bb1 proteins produced in *Bt* crops do not have a direct impact on the survival, development, growth, or fecundity of *P*. *pseudoannulata* and *Pirata subpiraticus* (also in the Lycosidae) [12,13] or *U*. *insecticeps* [14]. However, Zhou et al. [18] have found that enzyme activities in *Pardosa pseudoannulata* and *U*. *insecticeps* are significantly affected when they preyed upon fruit flies fed with Cry1Ab protein. Studies on *Bt* maize have focused on how Cry3Bb1 might affect the non-target web-building spider *Theridion impressum* but have found no negative influence when those spiders either prey upon arthropod species (mixed prey, lacewing, or corn rootworm) or are exposed to transgenic maize pollen via web re-ingestion [11]. Research by Ludy and Lang [9] has shown that the web-building *Araneus diadematus* (Araneae: Araneidae) is affected, but not detrimentally, when it ingests web-trapped pollen from Cry1Ab-expressing maize.

Although several *Bt* plant–herbivore–spider tritrophic bioassays have been performed, the way in which those crops that produce Cry1Ac proteins interact trophically with pests and spiders has not previously been studied in the laboratory. Here, we conducted a tritrophic bioassay of *Bt* cabbage (*Brassica oleraceae* var. *capitata*) that expresses this protein. This cabbage was developed to be resistant to the diamondback moth *Plutella xylostella* (Lepidoptera: Plutellidae), and the cabbage worm *Pieris rapae* (Lepidoptera: Pieridae) [19]. Both pests severely damage cabbage productivity and quality [20–21]. Our investigation focused on the wolf spider (*Pardosa astrigera* L. Koch). We considered this an appropriate test species because it is the dominant ground-dwelling spider in Korea. Moreover, it is an important natural enemy of *P*. *xylostella* on both cabbage and oilseed rape (*Brassica napus*) [22]. As a generalist predator, this spider is widely distributed throughout terrestrial environments, including agricultural lands in Korea, Japan, China, Taiwan, and Russia [23]. For our bioassays, we chose one fruit fly species, *Drosophila melanogaster*, because it is a primary consumer of cabbage and also a prey item for the wolf spider. As saprophytic insects, fruit flies are attracted to any crops, including cabbage, that provide fermenting tissue for their ovipositioning [24]. They utilize the fruits, flowers, and decaying materials of other plant parts [25], and have been observed for several years during our field experiments with cabbage [26]. We have previously confirmed that they are attracted to 7- to 14-day-old decaying cabbage tissues and can successfully reproduce on those tissues under laboratory conditions (26±1°C). They are relatively popular prey for Lycosid spiders in terrestrial environments, e.g., crop fields [27,28] and can be easily reared in a laboratory [29].

## Materials and Methods

### Plant materials

Seedlings of transgenic and non-transgenic cabbage lines were provided by the Biotechnology Institute of Nongwoo Bio Company Ltd., Korea. Insecticidal activity in transgenic Line C30 had been verified in the laboratory [19] and also observed in the field [29]. Plants of this line were derived from the inbred, non-transgenic Line AD126 and contained *cry1Ac1* under the control of the cauliflower mosaic virus 35S promoter and the *nos* terminator. Both genotypes were cultivated in an experimental field at the Korea Research Institute of Bioscience and Biotechnology (KRIBB), Cheongju, Chungcheongbuk-do, Korea (36°43′N, 127°26′E; elevation, 35 m) from April to November 2012. As estimated by Enzyme-Linked Immunosorbent Assays (ELISAs), Cry1Ac1 protein concentrations in these field-grown plants varied between 36 and 125 ng g^-1^ dry weight (DW), depending upon growth stage and sampling date (S1 Fig). We also measured the levels of Cry1Ac1 protein in leaves from transgenic cabbage that had been decaying at room temperature (RT; 26±1°C) in the laboratory for 7 to 14 d. Protein concentrations were 110, 114, and 80 ng g^-1^ DW after 1, 7, and 14 d, respectively (S2 Fig).

Conventional insecticides and fungicides were not sprayed during the study period. The harvested heads of transgenic and non-transgenic cabbages were placed in a freeze drier (Freezone 2.5; LABCONCO, USA), then ground into powders with a blender (7011HS; Waring, USA) before being stored at –20°C.

### Verification of insecticidal activity of *Bt*-drosophila media

To verify the persistence of insecticidal bioactivity in fruit fly-rearing media containing *Bt* cabbage powder, we performed bioassays using larval colonies of the target species, *Plutella xylostella*, that had been obtained from the Biotechnology Institute of Nongwoo Bio Company Ltd., Korea. In preparation, batches of culture media were made from cane sugar (18.0 g), corn meal (20.0 g), wheat meal (3.0 g), dry yeast (5.4 g), and agar (3.6 g) dissolved in 450 mL of distilled water. After the media were boiled and cooled to 50°C, either transgenic or non-transgenic cabbage powder (50 g) was added along with propionic acid (0.75 mL) and 4 mL of nipagin (100 g L^-1^ methyl 4-hydroxybenzoate in 95% ethanol). Afterward, 15-mL aliquots of these media were transferred to 30-mL drosophila bottles (Hansol Tech, Korea) that were then plugged with sponges. For the bioassays, three media treatment groups were used, based on how much time had elapsed since the powdered cabbage was added (1, 7, or 14 d; all stored at RT). Afterward, all groups were refrigerated at 4±1°C. Samples of the drosophila media were applied to the cabbage leaves, which were then placed on insect breeding dishes (Cat. No. 310050; SPL Life Sciences Co., Ltd. Korea). Finally, 10 *P*. *xylostella* larvae (second instar) were added to the dishes and held at RT. The media-treated leaves were replaced with fresh ones every 48 h, and the numbers of live or dead larvae were recorded at 24-h intervals for 7 d.

### The quality of *D*. *melanogaster* as prey

Fruit fly adults, obtained from Hansol Tech, were reared in drosophila bottles at RT, 60±5% relative humidity (RH), and a 16-h photoperiod. As parents, 30 pairs of *D*. *melanogaster* adults were transferred to sponge-plugged drosophila bottles (30-mL volume; Hansol Tech) that contained media supplemented with transgenic or non-transgenic cabbage powder (five replications each). The insects were maintained for 24 h. As the new generation reached adulthood, the dates were recorded and the mature insects were immediately collected and transferred to a deep freezer (–80±1°C) for 1 h. After each experimental group of adults was separated by gender, their body sizes and weights were recorded. The lengths of the thorax and wing were measured by using a dissecting microscope mounted with a digital camera (excope X3, Korea). To attain a minimum weight level on the digital balance, we measured five individuals together per gender group.

### Tritrophic study

Every two weeks, each group of *D*. *melanogaster* adults was transferred to new media containing either transgenic or non-transgenic cabbage powder. Four replicates of the drosophila media and fruit flies (200 individuals per powder treatment) were freeze-dried for the ELISAs.

To obtain young wolf spiders, we collected 20 adult females carrying egg sacs in the KRIBB experimental field on 2 August 2013. They were kept in sponge-plugged drosophila vials filled with nursery bed soil (10-mL volume, 22 mm in diameter, 92 mm tall; Hansol Tech). The RH was maintained at 60±5% and the vials were kept at RT under a 16-h photoperiod in the insect rearing room. We began with the third instar in tritrophic feeding trials that involved 60 spiders divided into two equal groups. The groups were supplied every 2 d with fruit flies that had fed on either *Bt* cabbage or non-*Bt* cabbage. As the spiders grew larger, the amount of prey was increased from three each in the third and fourth instars to five for the fifth through seventh instars. Feeding experiments were terminated when the spiders reached the adult stage after 74 d.

### Evaluation of spider life history traits and body size

The number of surviving spiders was tallied daily. We also determined their developmental times (i.e., how long they spent in each instar), based on the presence of exuvium. When the feeding trials were completed, carapace width (CW), carapace length (CL), and tibia length were measured under a dissecting microscope mounted with an ocular micrometer. The carapace index (CI) was calculated as CW/CL × 100. After fresh weights (FWs) were recorded, four replicates per treatment (six spiders each, with dead ones excluded) were freeze-dried in preparation for the ELISAs. The six spiders were pooled to obtain a sufficiently large sample for this analysis.

### ELISA procedure

Using transgenic and non-transgenic cabbage leaves sampled from the field and fermented for 7 and 14 d, we determined the levels of Cry1Ac1 protein in the experimental drosophila media, fruit flies, and wolf spiders via sandwich ELISA, with Cry1Ab/Cry1Ac protein-specific kits (Agdia Inc., USA). Freeze-dried samples of all tissues, both plant and animal, were ground in an auto mill (Tokken, Japan). After their DW values were determined, they were homogenized in 1×phosphate buffer saline-tween wash buffer (PBST, Agdia) at a ratio of 1:10 (w/v). The homogenized samples were centrifuged at 9425 *g* for 5 min before 100 μL of each supernatant was transferred to the test wells of ELISA plates to which the enzyme conjugate and RUB6 diluent mixture were added. The plates were incubated at 25°C for 2 h. Afterward, the primary sample and enzyme conjugate mixture were discarded and the remainder was quickly washed, seven times, with 1×PBST before 100 μL of TMB substrate solution was added to each well. The plates were then incubated for 20 min at RT. Optical density of the test wells was measured on a plate reader at 650 nm. The Cry1Ac1 concentration of each sample was estimated from a seven-point (0, 1, 5, 10, 50, 100 and 200 ng g^-1^) standard curve fitted to the optical density values of a *Bt*-Cry1Ac standard (Biosense, Norway). The standards were run on each plate of samples and the curves were fitted for each plate.

### Statistical analysis

The developmental time and body size of spiders exposed indirectly to either *Bt* cabbage or non-*Bt* cabbage were analyzed with Student’s *t*-tests. Sizes and weights of the fruit flies that were directly exposed to *Bt* cabbage or non-*Bt* cabbage were also analyzed with Student’s *t*-tests. Survival was assessed by the Kaplan-Meier method and significant differences between the two cabbage groups were examined by log-rank tests. All statistical analyses were performed using STATISTICA v 8.0 (Statsoft, USA).

## Results

### Insecticidal activity of *Bt* cabbage on target species

**Fig 1:**
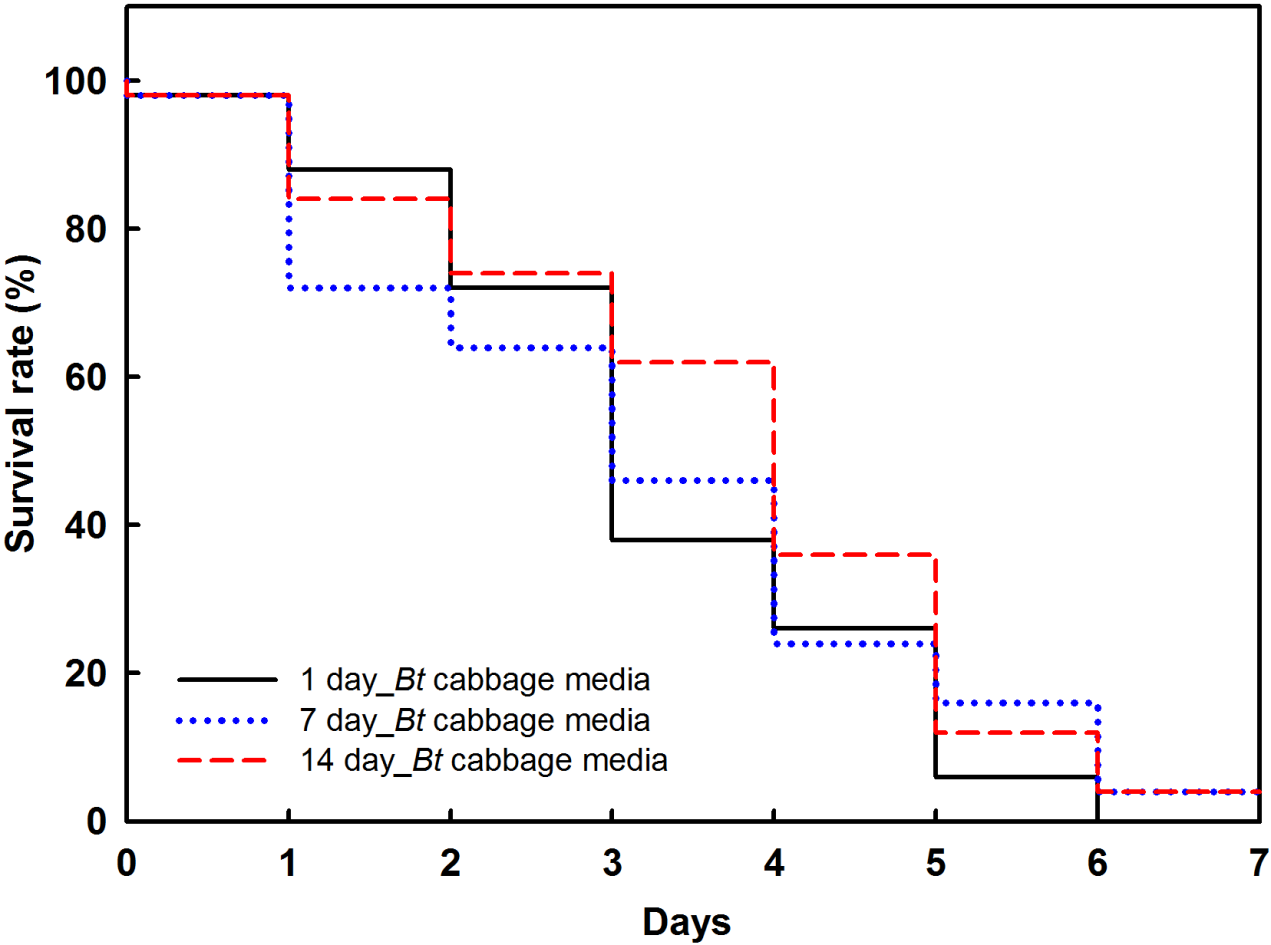
Overall survival of *P*. *xylostella* that had preyed upon drosophila media containing *Bt* cabbage powder. The drosophila medium was stored for 1 (black solid line), 7 (blue dotted line), or 14 (red dashed line) days at room temperature, and was used to verify its insecticidal activity. Data were derived from Kaplan-Meier curves (*n* = 10).

Over the 14-d observation period, survival rates for *P*. *xylostella* on media containing *Bt* cabbage powder declined relatively rapidly during the first 3 to 4 d, followed by a continuous decrease, such that most of the larvae from all treatment groups were dead by Day 7 (Fig 1).

### Effects of *Bt* cabbage on growth of *D*. *melanogaster*

The new generation of *D*. *melanogaster* reached adulthood after 9 d for both *Bt* and non-*Bt* cabbage media treatments. Overall, the females had larger body sizes and weights regardless of treatment group, although those differences were not significant (Table 1).

**Table 1:** Body size of *D*. *melanogaster* when exposed to media containing either non-*Bt* cabbage or *Bt* cabbage powder.

Factor	Non-*Bt*	*Bt*	*t*-value	Degrees of freedom	*P-*value^a^
Thorax length, male (mm) (*n* = 30)	0.7±0.01	0.7±0.01	1.400	58	0.167
Thorax length, female (mm) (*n* = 30)	0.9±0.01	0.9±0.01	0.872	58	0.387
Wing length, male (mm) (*n* = 30)	2.0±0.02	2.1±0.02	-1.831	58	0.072
Wing length, female (mm) (*n* = 30)	2.4±0.02	2.5±0.03	-0.615	58	0.541
Body weight, male (mg) (*n* = 10)	4.1±0.07	4.1±0.05	0.688	18	0.500
Body weight, female (mg) (*n* = 10)	6.2±0.1	6.1±0.06	1.044	18	0.310

Data are means±standard errors.

^a^*P*-values are from Student’s *t*-tests.

### Cry1Ac1 protein concentrations

**Fig 2:**
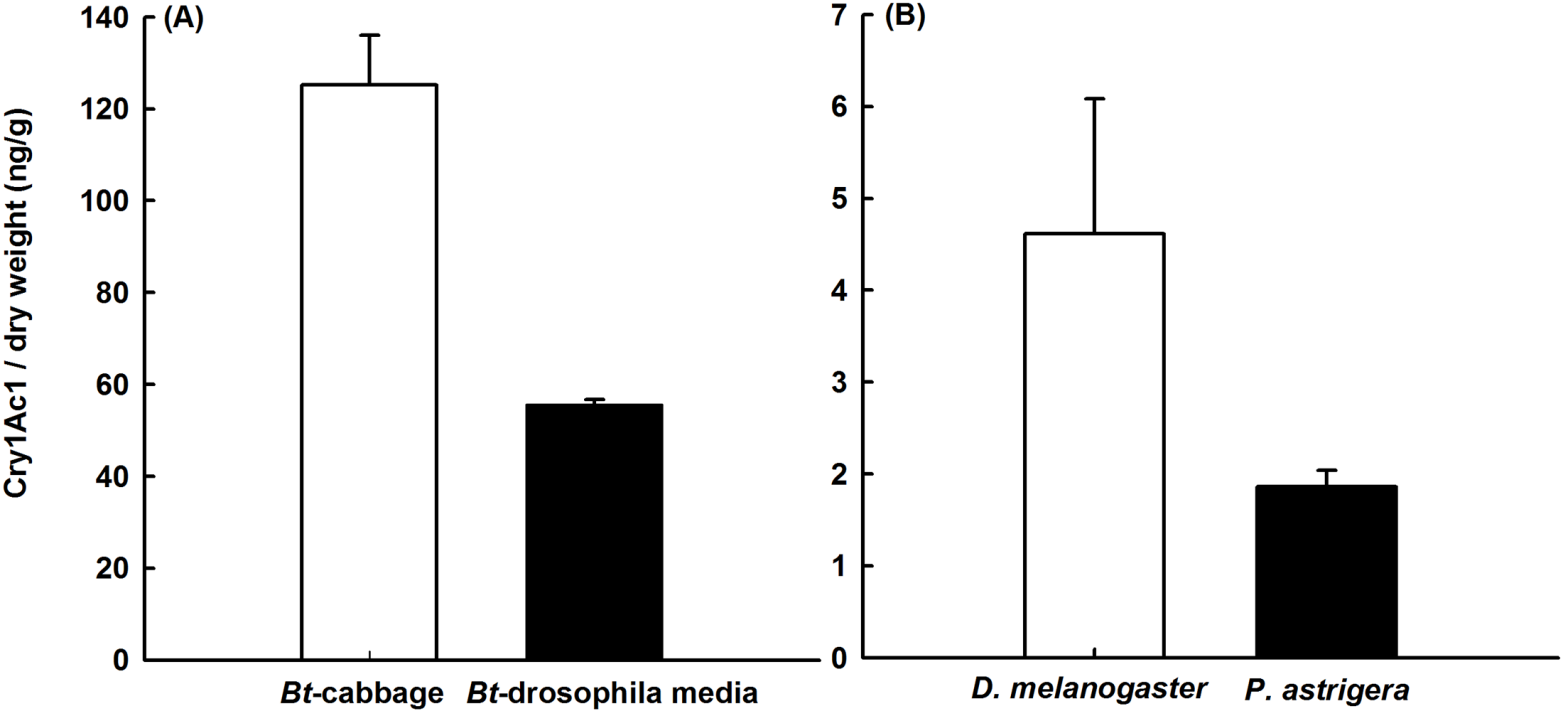
Cry1Ac1 concentrations (ng g^-1^ dry weight) in experimental samples. (A) *Bt* cabbage (*n* = 3) or drosophila media containing *Bt* cabbage (*n* = 4) and (B) *D*. *melanogaster* feeding on *Bt* cabbage (*n* = 4) or *P*. *astrigera* that had preyed upon *Bt* cabbage-fed *D*. *melanogaster* (*n* = 4). Data are means ± standard errors.

Cry1Ac1 concentrations in *Bt* cabbage leaf powder were 125.2 ng g^-1^ DW; protein levels in the medium containing transgenic-cabbage powder was 44% of the leaf concentration (Fig 2). The Cry1Ac1 concentration in fruit fly samples fed with *Bt* cabbage was 10.7% of the amount found on that *Bt* medium while the level in spiders exposed to fruit flies fed with transgenic cabbage was 3.2% of the amount measured from the *Bt* medium. No Cry1Ac1 protein was detected in either the fruit flies that fed on non-*Bt* media or the spiders that preyed upon them.

### Effects of *Bt* cabbage on spider life history traits and growth

**Fig 3:**
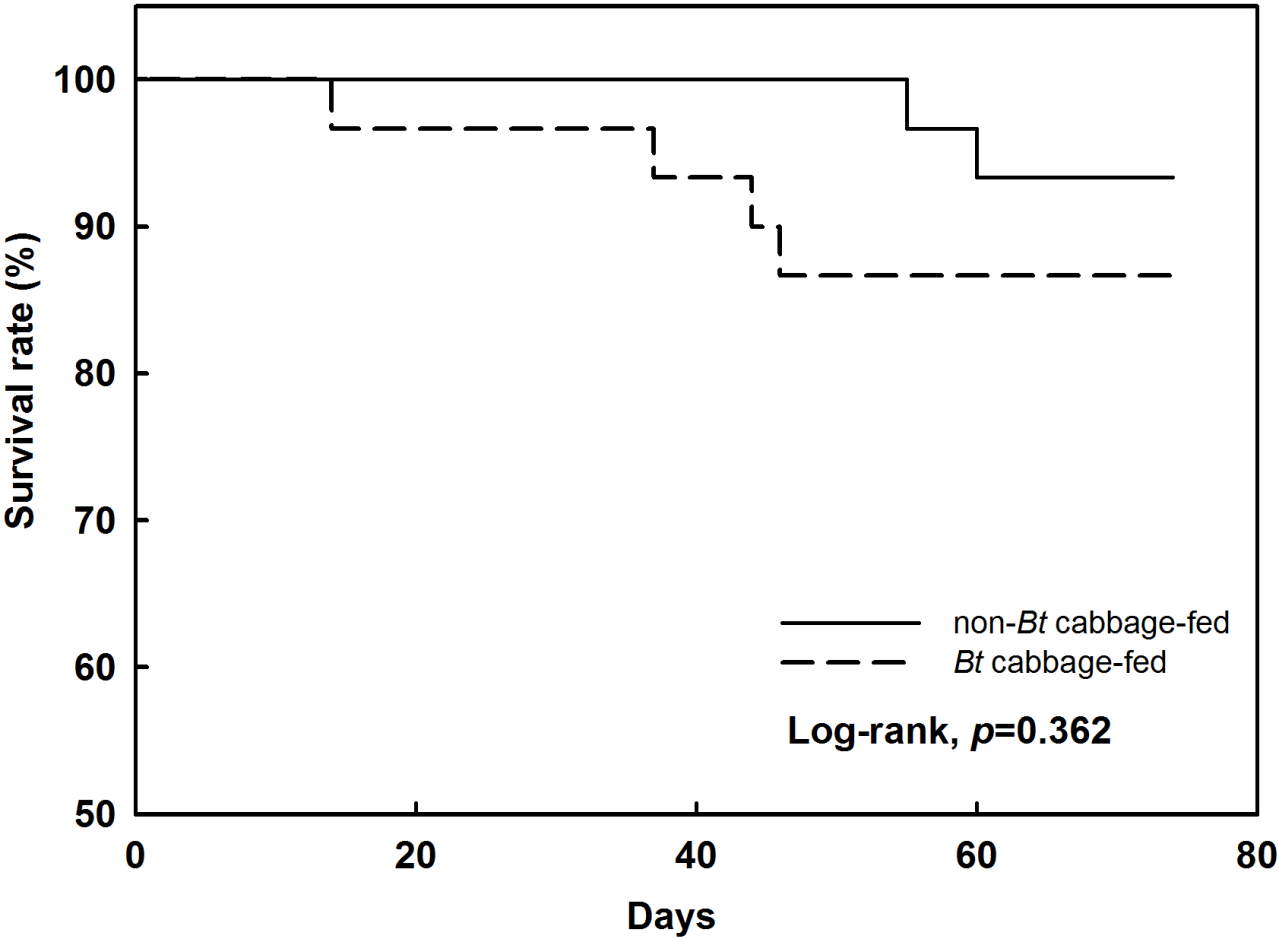
Overall survival of the wolf spider, *P*. *astrigera* that had preyed upon *D*. *melanogaster* fed with *Bt* or non-*Bt* cabbage. Data were derived from Kaplan-Meier curves (*n* = 30).

Survival rates for wolf spiders between the third instar and adulthood were not significantly affected by indirect exposure to *Bt* cabbage (Fig 3). In addition, preying upon fruit flies fed with transgenic cabbage did not affect the timing of each development stage or the entire life span (Table 2).

**Table 2:** Time (days) spent in each stage of development for *P*. *astrigera* from 3rd instar to adult emergence when exposed to either non-*Bt* cabbage-fed or *Bt* cabbage-fed *D*. *melanogaster*.

Developmental stage	Non-*Bt* (d)	*Bt* (d)	*t*-value	Degrees of freedom	*P-*value^a^
3rd instar	6.2±0.3	6.8±0.3	–1.438	57	0.156
4th instar	11.5±0.6	10.7±0.4	0.831	57	0.409
5th instar	11.9±0.4	12.1±0.5	–0.309	57	0.758
6th instar	16.3±0.7	15.0±0.7	1.219	55	0.228
7th instar	18.5±1.1	16.8±0.6	1.199	15	0.248
3rd instar to adult	52.1±1.0	50.1±1.2	1.589	54	0.118

Data are means±standard errors.

^a^*P*-values are from Student’s *t*-tests.

Overall, morphological traits (i.e., FW, carapace width and length, carapace index, and tibia length) were not significantly different among feeding groups (Table 3, *P*>0.05).

**Table 3:** Comparisons of body sizes for *P*. *astrigera* exposed to non-*Bt* cabbage-fed versus *Bt* cabbage-fed *D*. *melanogaster*.

Factor	Non-*Bt* (*n* = 28)	*Bt* (*n* = 26)	*t*-value	Degrees of freedom	*P-*value^b^
Fresh weight (mg)	23.8±0.6	24.5±0.8	–0.677	52	0.501
Carapace width (mm)	2.2±0.02	2.2±0.03	1.136	52	0.207
Carapace length (mm)	2.9±0.03	2.8±0.1	0.874	52	0.356
CI^a^	77.0±0.5	77.7±1.4	0.218	52	0.634
Tibia length (mm)	2.4±0.04	2.3±0.1	0.696	52	0.490

Data are means±standard errors.

^a^CI, carapace index was calculated as CI = CW/CL × 100

^b^*P*-values are from Student’s *t*-tests.

## Discussion

### Biotransfer of *Bt* protein via tritrophic interaction

In our study, the mean concentration of Cry1Ac1 protein in the fruit fly media was 56 ng g^-1^ DW, which was within the range of 36 to 125 ng g^-1^ DW measured from field-grown transgenic cabbage. Therefore, those media concentrations realistically represented what fruit flies are exposed to under field conditions.

After our 74-d feeding trials, we detected Cry1Ac1 protein in both the fruit fly and wolf spider samples, thereby demonstrating that the protein could be transferred from primary consumers to predators through the food chain. Such transfers via tritrophic interactions have also been described previously. For example, Zhou et al. [18], reported that the bio-transfer rates of Cry1Ab were 5% (5 ng mg^-1^ FW) for the fruit fly primary consumer and 18% (18 ng mg^-1^ FW) for the *P*. *pseudoannulata* predator when exposed either directly or indirectly to drosophila media containing 100 ng FW mL^-1^ of protein. All of these results suggested that Cry1Ab and Cry1Ac1 proteins do not accumulate in spider predators. Even though Cry protein levels in those food sources and the lengths of the test periods differed among these studies, the amounts of protein measured in the primary consumer and predators followed similar trends. Finally, Meissle and Romeis [30] have reported that, under both short-term (1–8 d) or long-term (28–64 d) feeding, bioaccumulations of Cry3Bb1 protein do not occur in the non-target spider *Phylloneta impressa* (Araneae: Theridiidae) or in its prey *Diabrotica virgifera virgifera* (Coleoptera: Chrysomelidae) and *Chrysoperla carnea* (Neuroptera: Chrysopidae).

### The effect of *Bt* crops on life history traits of spider predators

To date, *Bt* crops have not been shown to influence negatively the survival and growth of non-target spiders. The same was noted in our examination with *Bt* cabbage and wolf spiders. The morphological traits (body weight, carapace length and width, tibia length) were not significantly different between test groups in the present study. Among those traits, the carapace width has, in particular, been considered an important indicator of the growth of lycosid spiders [31–33].

Similar results have been described for web-building *Theridion impressum* and Cry3Bb1-expressing maize pollen under laboratory conditions [11], as well as the garden spider *Araneus diadematus* and Cry1Ab maize pollen [9]. In the case of ground-dwelling spiders, Tian et al. [12] have observed that the survival, development, and fecundity of *Pardosa pseudoannulata* are not significantly influenced when they prey upon planthoppers that were fed with Cry1Ab protein-expressing rice. Similarly, Chen et al. [13] have shown that the survival and fecundity of *Pirata subpiraticus* were not negatively affected when they preyed upon leafrollers fed with *Bt* (Cry1Ab) rice. Although they did find that developmental times were delayed for spiders indirectly exposed to the transgenic rice when compared with the control group, this may have been due to other reasons, such as the nutritional quality of the prey. In contrast, our study results demonstrated that *Bt* cabbage did not affect the growth of fruit flies, thereby making it unlikely that prey quality influenced the life history of these wolf spiders.

## Conclusions

Although numerous field studies have investigated how transgenic cotton and rice producing Cry1Ac proteins affect non-target arthropod communities [34–38], less attention has been focused on measuring *Bt* protein concentrations and examining trophically the consequences of *Bt* protein consumption [39,40]. Our tritrophic assay showed that young wolf spiders grew normally from the third instar to adulthood when reared on fruit flies that had been fed either *Bt* cabbage or non-*Bt* cabbage. Although we detected the presence of Cry1Ac1 protein in the spider bodies, it did not affect their development and survival. Thus, cultivation of transgenic *Bt* cabbage does not influence the survival and growth of wolf spiders.

## Supporting Information

S1 Fig Cry1Ac1 protein concentrations in leaves of field-grown *Bt* cabbage plants (Line C30) in Summer 2012 (A) and Autumn 2012 (B). Data are means and standard errors (*n* = 3).
(DOCX)

S2 Fig Cry1Ac1 concentrations in leaves of *Bt* cabbage (Line C30) decayed for 1, 7, or 14 d in the laboratory. Data are means and standard errors (*n* = 3).
(DOCX)
