# Supplementary material for: Transgenic Cabbage Expressing Cry1Ac1 Does Not Affect the Survival and Growth of the Wolf Spider, Pardosa astrigera L. Koch (Araneae: Lycosidae)
Source: PLoS One. 2016 Apr 7;11(4):e0153395. doi: 10.1371/journal.pone.0153395 (PMC4824485; doi:10.1371/journal.pone.0153395)
Supplement: S1 Fig — Data are means and standard errors (n = 3). (DOCX) [file pone.0153395.s001.docx]

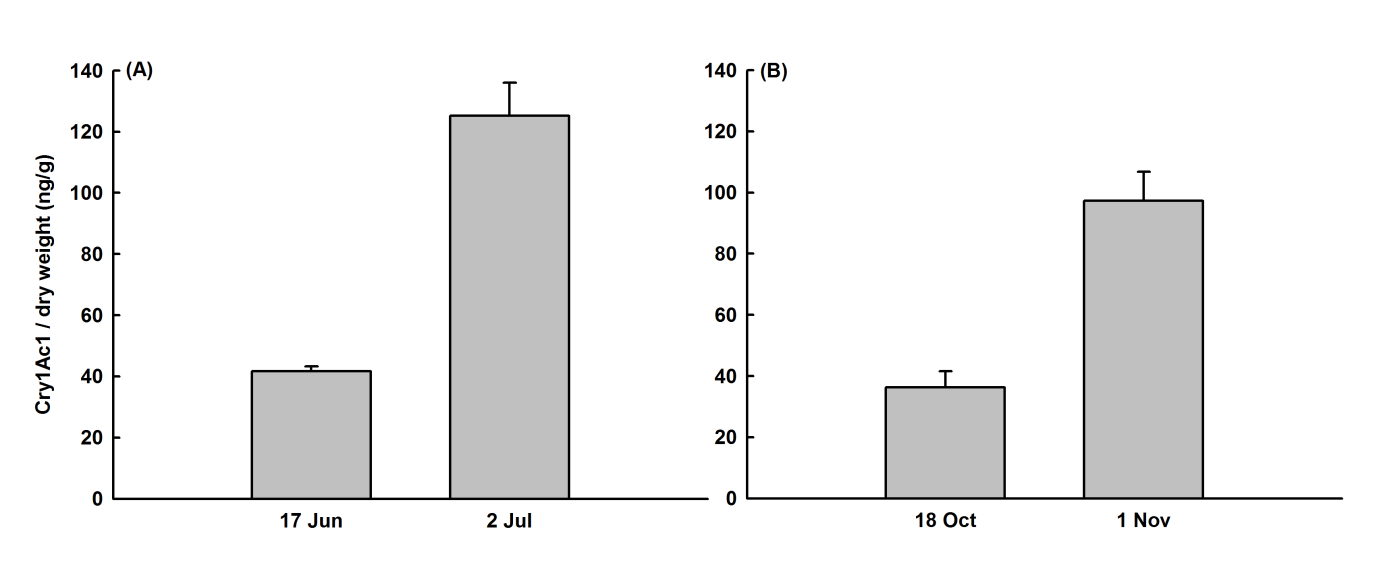


S1 Fig. Cry1Ac1 protein concentrations in leaves of field-grown *Bt* cabbage plants (Line C30) in Summer 2012 (A) and Autumn 2012 (B). Data are means and standard errors (*n*=3).
